# Incidence of Pneumococcal Pneumonia among Adults in Rural Thailand, 2006–2011: Implications for Pneumococcal Vaccine Considerations

**DOI:** 10.4269/ajtmh.15-0429

**Published:** 2015-12-09

**Authors:** Barameht Piralam, Sara M. Tomczyk, Julia C. Rhodes, Somsak Thamthitiwat, Christopher J. Gregory, Sonja J. Olsen, Prabda Praphasiri, Pongpun Sawatwong, Sathapana Naorat, Somrak Chantra, Peera Areerat, Cameron P. Hurst, Matthew R. Moore, Charung Muangchana, Henry C. Baggett

**Affiliations:** Nakhon Phanom Provincial Health Office, Nakhon Phanom Province, Thailand; Faculty of Public Health, Khon Kaen University, Khon Kaen Province, Thailand; Epidemic Intelligence Service, Division of Scientific Education and Professional Development, Centers for Disease Control and Prevention, Atlanta, Georgia; National Center for Immunization and Respiratory Diseases, Centers for Disease Control and Prevention, Atlanta, Georgia; International Emerging Infections Program, Global Disease Detection Center, Thailand Ministry of Public Health–United States Centers for Disease Control and Prevention Collaboration, Nonthaburi, Thailand; Division of Global Health Protection, Centers for Disease Control and Prevention, Atlanta, Georgia; Influenza Division, Centers for Disease Control and Prevention, Atlanta, Georgia; Influenza Program, Thailand Ministry of Public Health–United States Centers for Disease Control and Prevention Collaboration, Nonthaburi, Thailand; Sa Kaeo Provincial Health Office, Sa Kaeo Province, Thailand; National Vaccine Institute, Nonthaburi, Thailand; Faculty of Medicine, Khon Kaen University, Khon Kaen Province, Thailand

## Abstract

The incidence of pneumococcal pneumonia among adults is a key driver for the cost-effectiveness of pneumococcal conjugate vaccine used among children. We sought to obtain more accurate incidence estimates among adults by including results of pneumococcal urine antigen testing (UAT) from population-based pneumonia surveillance in two Thai provinces. Active surveillance from 2006 to 2011 identified acute lower respiratory infection (ALRI)–related hospital admissions. Adult cases of pneumococcal pneumonia were defined as hospitalized ALRI patients aged ≥ 18 years with isolation of *Streptococcus pneumoniae* from blood or with positive UAT. Among 39,525 adult ALRI patients, we identified 481 pneumococcal pneumonia cases (105 by blood culture, 376 by UAT only). Estimated incidence of pneumococcal pneumonia hospitalizations was 30.5 cases per 100,000 persons per year (2.2 and 28.3 cases per 100,000 persons per year by blood culture and UAT, respectively). Incidence varied between 22.7 in 2007 and 43.5 in 2010, and increased with age to over 150 per 100,000 persons per year among persons aged ≥ 70 years. Viral coinfections including influenza A/B, respiratory syncytial virus (RSV), and adenovirus occurred in 11% (44/409) of pneumococcal pneumonia cases tested. Use of UAT to identify cases of pneumococcal pneumonia among adults in rural Thailand substantially increases estimates of pneumococcal pneumonia burden, thereby informing cost-effectiveness analyses and vaccine policy decisions.

## Introduction

Pneumonia is a leading cause of morbidity and mortality worldwide,^1^ with an estimated 120 million episodes and 1.3 million fatalities among children.^2^,^3^ *Streptococcus pneumoniae* (pneumococcus) is the most common vaccine-preventable bacterial etiology of pneumonia, causing approximately 18% of cases in children globally.^2^ Pneumococcal conjugate vaccines (PCVs) are highly effective at preventing vaccine-type invasive pneumococcal disease (IPD) and radiographically confirmed pneumonia in children.^4^–^9^

Pneumococcal pneumonia is also a leading cause of morbidity and mortality among adults.^8^,^10^,^11^ PCV use among children prevents acquisition of vaccine-type pneumococci, reducing transmission to other children and adults.^12^,^13^ Most countries with widespread PCV use in children have seen substantially reduced rates of IPD and pneumonia among adults, a group not targeted for vaccination.^8^,^12^,^14^,^15^ Thus, herd effects among adults are the major drivers of cost-effectiveness estimates when considering PCV recommendations for children.

Estimates of pneumococcal pneumonia burden among adults in Asia are limited. In a 2009 review, annual IPD incidence among adults aged ≥ 65 years in Asia ranged from 7.7 to 74.0 cases per 100,000 persons per year with case fatality rates from 21.7% to 42.5% among published studies.^16^ However, most cases of pneumococcal pneumonia are not associated with bacteremia and not counted in IPD incidence estimates.^7^ Pneumococcal urine antigen testing (UAT) provides a complementary approach to detecting pneumococcal disease and estimating burden among adults. UAT has the ability to detect non-bacteremic and bacteremic pneumococcal pneumonia and is 70–100% sensitive (70–80% in most studies) and 67–100% specific (80 to > 90% in most studies).^17^–^23^

To inform PCV policy discussions in Thailand and southeast Asia, we aimed to improve the estimation of pneumococcal pneumonia incidence among adults in Thailand by systematically using UAT in pneumonia surveillance. In addition, we explored the temporal relationship between pneumococcal pneumonia incidence and viral coinfections to assess the potential impact of other interventions such as influenza vaccines.

## Materials and Methods

### Study population.

In collaboration with the U.S. Centers for Disease Control and Prevention (CDC) International Emerging Infections Program, the Thailand Ministry of Public Health conducted active, population-based surveillance for pneumonia hospitalizations at all 20 acute care hospitals in two rural provinces between 2002 and 2014.^24^ Sa Kaeo Province, with a 2010 population of 555,471, borders Cambodia in eastern Thailand. Nakhon Phanom Province, with a 2010 population of 583,264, borders Laos in northeastern Thailand.^25^ Provincial population estimates for 2010–2011 were available from the 2010 National Economic and Social Development Board (NESDB) of Thailand.^26^ For 2006–2009, the 2010 NESBD age distribution was applied to the 2006–2009 NESDB provincial population estimates to account for inconsistencies between the 2010 and previous provincial population estimates. PCV is available in the private market in both provinces, but uptake is minimal; a recent case–control pneumonia study in children aged < 5 years found that none of the 641 community-based controls had received PCV (unpublished data).

### Acute lower respiratory infection surveillance.

**Figure 1.**
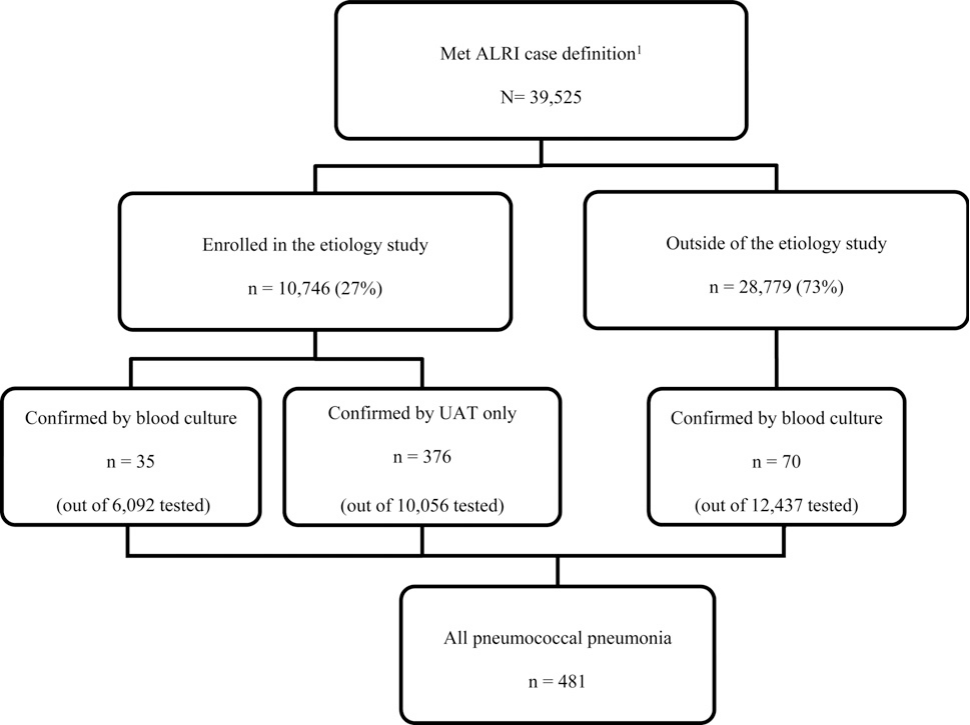
Flowchart of surveillance and etiology study participants among adults aged ≥ 18 years hospitalized with acute lower respiratory infection (ALRI) in rural Thailand, 2006–2011.^1^ Patients meeting ALRI case definition and included in the pneumonia surveillance system.

Surveillance staff reviewed hospital admissions daily to identify hospitalizations consistent with acute lower respiratory infection (ALRI). A case of ALRI was defined as ≥ 1 sign/symptom of active infection (reported fever, measured temperature > 38.2°C or < 35°C, chills, or an abnormal white blood cell count [> 11,000 or < 3,000 cells/mL]) and ≥ 1 respiratory sign or symptom (abnormal breath sounds, tachypnea, cough, chest pain, hemoptysis, sputum production, or dyspnea) in a hospitalized patient who had lived ≥ 6 months in one of the study provinces. Patients hospitalized with ALRI more than once within 14 days were counted as a single case. From 2002 to 2007, patients meeting the ALRI case definition who had a chest radiograph performed within 48 hours post-admission were eligible for enrollment in an etiology study. From 2008 to 2012, every other ALRI case with or without a chest radiograph was systematically sampled by ward and admission time for enrollment. Radiographs were digitized and interpreted by a panel of radiologists using standard criteria as previously described.^27^,^28^ An adult case of pneumococcal pneumonia was defined as a hospitalized ALRI patient ≥ 18 years and either isolation of *S*. *pneumoniae* by blood culture or a positive UAT (Figure 1

). We assumed that patients hospitalized with ALRI and laboratory-confirmed pneumococcal infection had pneumonia, regardless of radiographic findings.

### Specimen collection and testing.

Urine and nasopharyngeal specimens were collected at enrollment from all consenting patients in the etiology study. Urine specimens were stored at 4–8°C before transport within 48 hours to the provincial hospital laboratories for pneumococcal antigen testing using Binax NOW^®^ UAT (Binax, Portland, ME); positive results were reported to clinicians within 12 hours. Nasopharyngeal swab specimens were collected using polyester swabs (Puritan^®^,Guilford, ME) through July 2010 and flocked swabs (FLOC Swabs^™^, Copan, Murrieta, CA) thereafter. These specimens were stored at 4–8°C for < 24 hours before being frozen at −70°C and transported weekly on dry ice to Thailand's National Institutes of Health where they were tested for influenza A/B virus, respiratory syncytial virus (RSV), and adenovirus by real-time reverse transcription polymerase chain reaction.^29^

Blood cultures were collected as clinically indicated, regardless of etiology study enrollment.^30^ Blood samples were transported to the provincial hospital laboratories at 15–30°C within 24 hours and processed using BacT/ALERT^®^ 3D microbial detection system (bioMeriux, Hazelwood, MO) as previously described.^31^ Antibiotic use before blood culture was determined by testing for serum antibiotic activity by disc assay.^30^–^32^

### Ethical statement.

Informed consent was obtained from patients in the etiology study according to the protocol approved by a CDC Institutional Review Board (no. 3754) and the Thailand Ministry of Public Health Ethical Review Committee.

### Statistical analysis.

The analysis period was January 2006 to December 2011. We compared characteristics of UAT-positive versus UAT-negative ALRI patients using χ^2^ or F tests. We calculated age-specific incidence of pneumococcal pneumonia by dividing the number of cases in each age group by the adult population of the two provinces.^33^

To account for ALRI patients not undergoing UAT, we adjusted the age-specific incidence for the sampling frame and non-enrollment of eligible ALRI patients. We assumed that the proportion of ALRI patients positive by UAT in the etiology study was the same as the proportion positive among hospitalized ALRI patients who did not enroll in the etiology study. Thus, the proportion of enrolled patients that were positive by UAT was multiplied by the total number of eligible ALRI patients within each age group to calculate an adjusted numerator for the incidence estimates. Incidence adjustments for 2006–2007 were stratified by those with or without a chest radiograph, because only ALRI patients with a chest radiograph were enrolled in the etiology study during this period. For ALRI patients without a chest radiograph, who did not enroll in the etiology study in 2006–2007 (not eligible in those years), the average UAT percent positivity among those without a chest radiograph from 2008 to 2011 was used for the incidence estimates. The incidence adjustment applied to UAT cases only; we did not adjust incidence attributable to blood culture–confirmed cases.

We also present monthly pneumococcal pneumonia case counts and influenza A/B, RSV, and adenovirus percent positivity to explore pneumococcal seasonality and correlation with viral infections.

## Results

**Figure 2.**
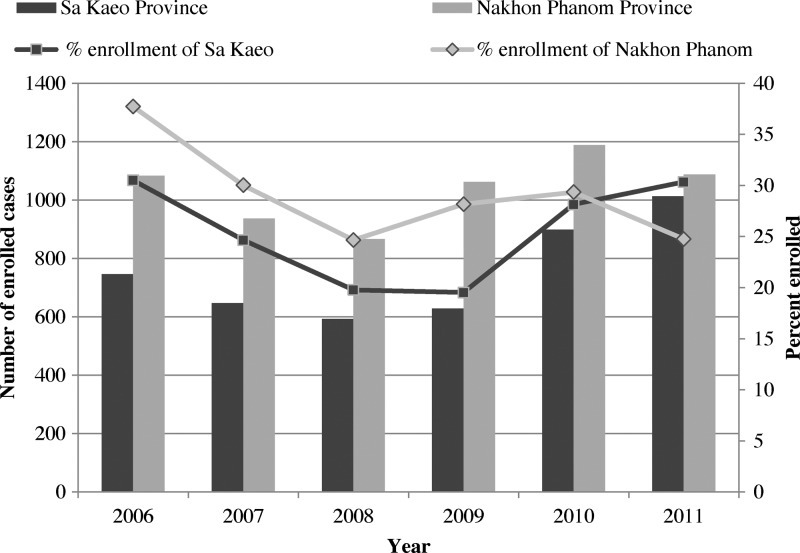
Number and percent of eligible cases enrolled among acute lower respiratory infection (ALRI) patients by province and year in rural Thailand, 2006–2011.

From January 2006 to December 2011, 39,525 adults were hospitalized with ALRI and 2,276 (5.8%) died. On the basis of sampling methods, 21,668 patients were eligible for enrollment in the etiology study and 10,746 enrolled (50% of eligible, 27% of all ALRI). The number and proportion of enrolled patients was higher in Nakhon Phanom than in Sa Kaeo for most years (Figure 2

). Non-enrolled patients were more likely to require mechanical ventilation (12% versus 6.6%) and die (6.7% versus 3.2%). The proportion with radiographically confirmed pneumonia and with comorbid conditions was similar between groups (data not shown).

Among 10,746 ALRI patients enrolled in the etiology study, 411 (3.8%) had laboratory-confirmed pneumococcal pneumonia, including 35 confirmed by blood culture (among 6,093 tested) and 376 by UAT only (among 10,056 tested); 20 (4.9%) of 411 were confirmed by both tests. When limited to the 6,093 ALRI patients with blood culture and UAT performed, 35 had pneumococcal pneumonia confirmed by blood culture while 225 had positive UAT only, resulting in 6.4 cases of non-bacteremic pneumococcal pneumonia for every bacteremic case. Among 28,779 (73%) patients with ALRI not enrolled in the etiology study, 12,442 patients had blood cultures performed and 70 had culture-confirmed pneumococcal pneumonia, resulting in a total of 481 laboratory-confirmed pneumococcal pneumonia cases among those hospitalized with ALRI (*N* = 39,525) (Figure 1).

Of pneumococcal pneumonia patients, 40% (*N* = 193) were ≥ 70 years of age and 52% (*N* = 249) were male (Table 1). Clinical characteristics most commonly reported included cough (91%) and fever > 38.2°C (89%). Of the cases, 380 (79%) had a chest radiograph, of which 74% (*N* = 282) had evidence of radiographic pneumonia. Comorbidities most commonly reported included heart disease (10%) and human immunodeficiency virus infection (7%). Eighty patients (17%) required mechanical ventilation and 52 (11%) died in the hospital. Among the 481 laboratory-confirmed cases of pneumococcal pneumonia, few had clinician discharge diagnoses (International Statistical Classification of Diseases and Related Health Problems, 10th edition; ICD-10) of bacterial pneumonia (*N* = 40) or *S*. *pneumoniae* infection (*N* = 23). Of 368 discharged cases, 19 (5.0%) were readmitted within 14 days for pneumonia-related causes and three of these died during their readmission.

Among 10,056 ALRI patients with UAT performed, 396 (3.9%) tested positive and 9,660 were negative (Table 1). UAT-positive patients were significantly more likely than UAT-negative patients to be aged ≥ 60 years, to have evidence of radiographic pneumonia, a smoking history, severe disease (mechanical ventilation, death), and discharge diagnoses consistent with pneumonia (*P* < 0.05). Of all patients with laboratory-confirmed pneumococcal pneumonia, those with blood culture–confirmed disease (*N* = 105) were significantly more likely than those confirmed by UAT only (*N* = 376) to have a history of liver disease (11% versus 1%) or smoking (40% versus 24%), to have evidence of radiographic pneumonia (90% versus 70%), and to be discharged with a diagnosis of *S*. *pneumoniae* (18% versus 1%) or septicemia (19% versus 3%) (*P* < 0.05). Cases confirmed by blood culture were also significantly more likely to be intubated (45% versus 9%) or to die (31% versus 5%) as compared with those confirmed by UAT only (*P* < 0.05). No culture-confirmed cases had blood collected for culture ≥ 2 days post-admission, suggesting that these infections were not nosocomial. Among patients with serum antibiotic activity testing, significantly more with blood cultures without pneumococcus detected had received pre-culture antibiotics (4,358/16,645 [26%]) compared with those with blood cultures positive for pneumococcus (4/89 [5%]) (*P* < 0.0001). The proportion of patients with positive UAT who had received pre-culture antibiotics (51/223 [23%]) was similar to those with negative UAT (1,235/4,949 [25%]) (*P* = 0.48).

**Figure 3.**
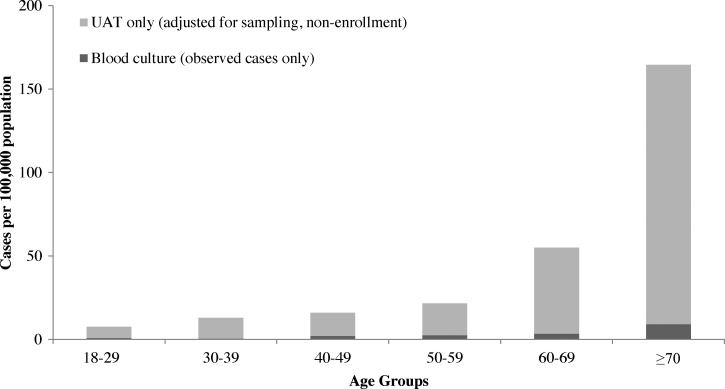
Incidence of pneumococcal pneumonia hospitalizations by age group among adults in rural Thailand, 2006–2011. UAT = urine antigen test.

The observed 481 cases of laboratory-confirmed pneumococcal pneumonia correspond to 10.1 adult pneumococcal pneumonia hospitalizations per 100,000 persons per year in these provinces from 2006 to 2011. After adjusting for the sampling frame and non-enrollment of eligible ALRI patients, the incidence rate of adult pneumococcal pneumonia hospitalizations was 30.5 cases per 100,000 persons per year, including 2.2 cases confirmed by blood culture and 28.3 cases confirmed by UAT alone. Incidence increased with age from 18–29 years to ≥ 70 years for both culture-confirmed cases (0.8–9.1 cases per 100,000 persons per year) and for cases overall (7.5–164.6 cases per 100,000 persons per year) (Figure 3

). Adjusted incidence (cases per 100,000 persons per year) varied by province (35.9 in Nakhon Phanom versus 24.7 in Sa Kaeo) and by year (27.8 in 2006, 22.7 in 2007, 23.0 in 2008, 32.4 in 2009, 43.4 in 2010, and 34.0 in 2011).

To explore the potential impact of UAT test performance on incidence estimates, we recalculated overall adjusted incidence using a range of reported sensitivities and specificities.^17^–^23^,^34^ When assuming the lowest reported sensitivity (70%)^34^ and specificity of 100%, the incidence would have been 41.3 cases per 100,000 persons per year. When assuming the lowest specificity (67%)^23^ and sensitivity of 100%, the incidence would have been 19.4 cases per 100,000 persons per year.

**Figure 4.**
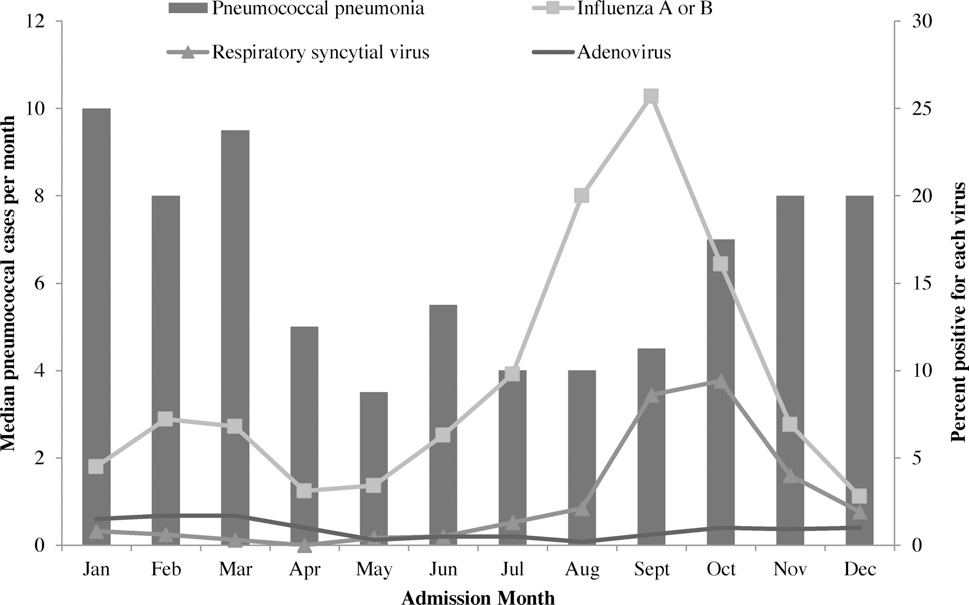
Median monthly number of pneumococcal pneumonia cases and viral percent positivity among hospitalized adults with acute lower respiratory infection (ALRI) in rural Thailand, 2006–2011.

Viral coinfections including influenza A/B, RSV, and adenovirus occurred in 11% (44/409) of pneumococcal pneumonia cases tested; however, viral infection, including influenza, was not more common among pneumococcal pneumonia cases than among ALRI patients without evidence of pneumococcal infection (Table 1). Median monthly case counts of pneumococcal pneumonia were higher in October–March (8.4) compared with April–September (4.4) (*P* < 0.03) for all years (Figure 4

). In contrast, percent positivity of influenza A/B and RSV was highest during August–October (Figure 4).

## Discussion

By systematically incorporating the more sensitive UAT assay into active, population-based ALRI surveillance in Thailand, we documented an annual rate of pneumococcal pneumonia hospitalizations among adults (30.5 cases per 100,000 persons per year) that was 14-fold higher than estimates from blood culture alone (2.2 cases per 100,000 persons per year). The highest rates were observed in persons aged ≥ 70 years. Despite this disease burden, health-care providers seldom diagnosed bacterial pneumonia or *S*. *pneumoniae*. A recent meta-analysis of diagnostic techniques for pneumococcal pneumonia in adults estimated that there are at least three additional non-bacteremic pneumococcal pneumonia cases for every bacteremic pneumonia case,^35^ whereas we found more than six non-bacteremic cases per bacteremic case.

A positive UAT in the absence of a positive blood culture may be due to the ability of UAT to detect less severe cases with lower bacterial load missed by blood culture.^22^ Blood culture sensitivity may also be more affected by pre-culture antibiotic use. We found that pre-culture antibiotic receipt was significantly more common among patients without pneumococcus detected by blood culture compared with those with positive cultures. Pre-culture antibiotic use has been reported to reduce estimates of pneumococcal bacteremia by 32–67%.^31^,^35^ We did not find evidence of reduced UAT positivity with prior antibiotic use, which differs from the findings of Said and others,^35^ who estimated a 26% reduction.

Data on pneumococcal disease incidence in Asia are limited. A previous study in Thailand estimated the incidence of pneumococcal bacteremia hospitalizations among adults aged ≥ 65 years to be 14.2 cases per 100,000 persons per year.^32^ One study in Taiwan found IPD incidence in adults ranged from 1.1 to 14.0 cases per 100,000 population.^36^ Published laboratory results from the Hong Kong reported 7.7 cases of IPD per 100,000 persons per year among adults aged ≥ 65 years.^37^ These published reports of IPD incidence provide limited basis for comparison to our results because most cases of pneumococcal pneumonia are not associated with bacteremia. A study in Singapore estimated incidence of pneumococcal pneumonia by hospital discharge diagnoses as 4.5 cases per 100,000 persons per year among adults aged 15–64 years, 32.2 cases per 100,000 persons per year among adults aged 65–74 years, and 94.8 cases per 100,000 persons per year among adults aged ≥ 75 years.^38^ These data lack laboratory confirmation and rely on physician diagnosis, likely resulting in underestimates of the incidence of pneumococcal pneumonia, which may explain why they differ so much from rates that we have estimated in Thailand.

Concurrent viral infections ranged from 1% (adenovirus) to 7% (influenza) of pneumococcal pneumonia cases. In the United States, modeled data from 1995 to 2006 showed that influenza may cause a modest increase in pneumococcal pneumonia between 5% and 6% of invasive pneumococcal pneumonia was associated with influenza infection.^39^ In the United States and other temperate climates, pneumococcal and influenza seasons are temporally correlated and peaks of both are observed in November–March.^40^ However, we found that peaks in influenza and RSV did not coincide with increases in median monthly cases of pneumococcal pneumonia in rural Thailand. Viral coinfections were not more common among pneumococcal cases compared with viral infections in ALRI patients without pneumococcal infection. These findings point to the importance of factors other than circulating influenza or RSV to account for seasonal patterns of pneumococcal disease in this setting.

There are several limitations to our study. Although a marked improvement over blood culture, reported UAT sensitivity ranges from 70% to 80%, suggesting that we likely missed pneumococcal cases and possibly underestimated incidence.^17^–^23^,^34^ Although UAT specificity is generally high, false positives also may have occurred^17^–^23^,^34^; the duration of UAT positivity after pneumococcal infection is unclear but has been documented for up to 6 weeks, so a positive UAT could represent past rather than present infection.^19^ In a sensitivity analysis, applying the lowest reported UAT sensitivity (70%)^34^ and specificity (67%),^23^ pneumococcal pneumonia incidence ranged from 19.4 to 41.3 cases per 100,000 persons per year.^17^–^23^,^34^ Only 50% of patients selected for potential enrollment (27% of all ALRI patients) were enrolled in the study. Non-enrollment was partially due to lack of study staff on weekends and difficulties enrolling severely ill patients, both of which could have biased our results. Our incidence calculations relied on the assumption that UAT percent positivity among those tested in the study was similar to those who met the ALRI case definition but were not enrolled and tested. Compared with ARLI patients enrolled in the etiology study, those that did not enroll were more likely to require mechanical ventilation or die, which could have resulted in underestimated pneumococcal pneumonia incidence, given the association of UAT positivity and disease severity.

Our findings offer a more accurate estimate of pneumococcal pneumonia incidence among adults in Thailand, which is critical for informing vaccine policy discussions and cost-effectiveness analyses surrounding PCV introduction. A previous cost-effectiveness analysis of PCVs in Thailand relied on passive national surveillance for adult pneumococcal pneumonia incidence. At the time, the available data found two cases per 100,000 persons per year among persons aged 15–24 years up to 40 cases per 100,000 persons per year among persons aged ≥ 65 years.^41^ Our estimates were substantially higher, ranging from 7.5 to 164.6 cases per 100,000 persons per year among adults aged 18–29 years to ≥ 70 years, respectively. In addition to the contribution of UAT, we identified patients for testing through active ALRI surveillance, providing more robust estimates of disease burden compared with passive surveillance.^41^ PCV is rarely used in Thailand; our estimates suggest a substantially greater opportunity for PCV's indirect effects (i.e., prevention of disease in adults by vaccinating children) in Thailand than previously estimated. Furthermore, recent data from a large randomized placebo-controlled trial (CAPiTA trial) among adults ≥ 65, which also used a UAT (different assay) for case detection, found approximately 46% efficacy of PCV13 against vaccine-type pneumococcal pneumonia, which shows the potential direct benefit of PCV13 in the prevention of pneumococcal pneumonia among adults in addition to the indirect benefits to adults from vaccinating children.^42^ Incidence estimates incorporating UAT could be used in future PCV cost-effectiveness analyses and inform vaccine policy decisions in Thailand and throughout southeast Asia.

## Figures and Tables

**Table 1:** Characteristics of adults hospitalized with ALRI with and without pneumococcal pneumonia based on blood culture or UAT in rural Thailand, 2006–2011

	Laboratory-confirmed pneumococcal pneumonia* (*N* = 481)	All UAT+ (*N* = 396)	All UAT− (*N* = 9,660)	*P* value†
Age (years), *n* (%)				
18–29	24 (5.0)	19 (4.8)	735 (7.6)	**0.004**
30–39	39 (8.1)	37 (9.3)	922 (9.5)	
40–49	61 (12.6)	43 (10.9)	1,206 (12.5)	
50–59	65 (13.5)	50 (12.6)	1,655 (17.1)	
60–69	99 (20.6)	84 (21.2)	1,977 (20.5)	
≥ 70	193 (40.1)	163 (41.2)	3,165 (32.8)	
Male sex, *n* (%)	249 (51.8)	202 (51.0)	5,031 (52.1)	0.68
Clinical characteristics, *n* (%)				
Fever (> 38.2°C or reported)	429 (89.2)	355 (89.7)	8,088 (83.7)	**0.002**
Abnormal WBC (> 11,000/mL or < 3,000/mL)	277 (62.5)	221 (61.2)	3,826 (43.8)	< **0.0001**
Cough	439 (91.3)	372 (93.9)	8,692 (90.0)	**0.001**
Sputum production	294 (61.1)	249 (62.9)	5,232 (54.2)	**0.001**
Hemoptysis	19 (4.0)	18 (4.6)	426 (4.4)	0.90
Chest pain	70 (14.6)	62 (15.8)	938 (9.7)	< **0.0001**
Dyspnea	338 (70.3)	276 (69.7)	5,306 (55.0)	< **0.0001**
Abnormal breath sounds	337 (70.1)	280 (70.7)	5,456 (56.5)	< **0.0001**
Tachypnea (based on clinical assessment)	215 (44.7)	164 (41.4)	3,396 (35.2)	**0.01**
Rales/crepitation‡	246 (73.0)	199 (71.1)	2,972 (54.4)	< **0.0001**
Rhonchi‡	105 (31.2)	88 (31.4)	1,524 (27.9)	0.20
Viral infections, *n* (%)‡				
Adenovirus‡	3/409 (0.8)	2/394 (0.5)	91/9,648 (0.9)	0.38
Respiratory syncytial virus‡	11/405 (2.7)	11/402 (2.8)	274/9,930 (2.9)	0.95
Influenza A/B viruses‡	30/405 (7.4)	30/390 (7.7)	971/9,518 (10.2)	0.11
Radiographic pneumonia, *n* (%)‡	282/380 (74.2)	228/318 (71.7)	3,654/6,855 (53.3)	< **0.0001**
Medical history, *n* (%)				
Any comorbid condition	128 (26.6)	101 (25.5)	2,375 (24.6)	0.68
Cancer	10 (2.1)	7 (1.8)	152 (1.6)	0.76
Liver disease	16 (3.3)	6 (1.5)	114 (1.2)	0.55
Renal disease	32 (6.7)	27 (6.8)	510 (5.3)	0.18
Heart disease	47 (9.8)	38 (9.6)	1,283 (13.3)	**0.03**
HIV	34 (7.1)	29 (7.3)	537 (5.6)	0.14
Smoking	133 (27.7)	101 (25.5)	2,056 (21.3)	**0.04**
Severity, *n* (%)				
O_2_ requirement§	447 (92.9)	363 (91.7)	8,775 (90.8)	0.57
Mechanical ventilation	80 (16.6)	39 (9.9)	591 (6.1)	**0.003**
Outcomes, *n* (%)				
Death	52 (10.8)	25 (6.3)	261 (2.7)	**0.0002**
Transferred/referred	39 (8.1)	29 (7.3)	792 (8.2)	
Discharge	368 (76.5)	329 (83.1)	8,402 (87.0)	
Self-discharge	22 (4.6)	13 (3.3)	197 (2.0)	
Discharge diagnoses, *n* (%)				
Consistent with any pneumonia	235 (48.9)	189 (47.7)	2,397 (24.8)	< **0.0001**
Bacterial pneumonia, unspecified	40 (8.3)	34 (8.6)	481 (5.0)	**0.001**
Pneumonia due to *Streptococcus pneumoniae*	23 (4.8)	8 (2.0)	20 (0.2)	< **0.0001**
Viral pneumonia, unspecified	1 (0.2)	1 (0.3)	29 (0.3)	0.86
Other pneumonia diagnoses∥	171 (35.6)	146 (36.9)	1,867 (19.4)	< **0.0001**
Septicemia	32 (6.7)	15 (3.8)	371 (3.8)	0.96

ALRI = acute lower respiratory infection; HIV = human immunodeficiency virus; UAT = urine antigen test; WBC = white blood cell.

* Among 481 pneumococcal pneumonia cases, 105 were confirmed by blood culture and 376 were confirmed by UAT only.

† Categorical variables tested by χ^2^ tests and continuous variables tested by F tests between UAT+ and UAT−. Bold font denotes statistical significance (*P* < 0.05).

‡ Among those tested.

§ Oxygen saturation < 92% or received oxygen supplementation.

∥ Other pneumonia diagnoses included: pneumonia due to other infectious organisms, pneumonia due to *Haemophilus influenzae*, pneumonia unspecified, and pneumonia in diseases classified elsewhere.
